# Patient Experiences and Challenges in the Management of Autoinflammatory Diseases—Data from the International FMF & AID Global Association Survey

**DOI:** 10.3390/jcm13051199

**Published:** 2024-02-20

**Authors:** Jürgen Rech, Georg Schett, Abdurrahman Tufan, Jasmin B. Kuemmerle-Deschner, Seza Özen, Koray Tascilar, Leonie Geck, Tobias Krickau, Ellen Cohen, Tatjana Welzel, Marcus Kuehn, Malena Vetterli

**Affiliations:** 1 Department of Internal Medicine 3, Rheumatology and Immunology, Friedrich-Alexander University (FAU), Erlangen-Nürnberg and Universitätsklinikum Erlangen, 91054 Erlangen, Germany; georg.schett@uk-erlangen.de (G.S.); koray.tascilar@uk-erlangen.de (K.T.); leonie.geck@fau.de (L.G.); 2 Deutsches Zentrum Immuntherapie, Friedrich-Alexander University (FAU), Erlangen-Nürnberg and Universitätsklinikum Erlangen, 91054 Erlangen, Germany; tobias.krickau@uk-erlangen.de; 3 Center for Rare Diseases Erlangen (ZSEER), Friedrich-Alexander University (FAU), Erlangen-Nürnberg and Universitätsklinikum Erlangen, 91054 Erlangen, Germany; 4 Division of Rheumatology, Department of Internal Medicine, Gazi University Ankara, 06560 Ankara, Turkey; atufan@gazi.edu.tr; 5 Division of Pediatric Rheumatology, Autoinflammation Reference Center Tübingen, Department of Pediatrics, University Hospital Tübingen, 72016 Tübingen, Germany; kuemmerle.deschner@uni-tuebingen.de; 6 Department of Pediatric Rheumatology, Hacettepe University, 06100 Ankara, Turkey; sezaozen@gmail.com; 7 Department of Paediatrics, Friedrich-Alexander University (FAU), Erlangen-Nürnberg and Universitätsklinikum Erlangen, 91054 Erlangen, Germany; 8 FMF & AID Global Association, 8306 Zurich, Switzerland; ecohen@verizon.net (E.C.); info@fmfandaid.org (M.V.); 9 Pediatric Rheumatology, University Children’s Hospital Basel (UKBB), University of Basel, 4001 Basel, Switzerland; tatjana.welzel@ukbb.ch; 10 Löwenpraxis, 6004 Luzern, Switzerland; kuehn.allergie@hin.ch

**Keywords:** autoinflammatory diseases, familial Mediterranean fever (FMF), Behcet’s disease, diagnosis, misdiagnoses, patient survey, pain, fatigue

## Abstract

Background: Autoinflammatory diseases (AIDs) are rare, mostly genetic diseases that affect the innate immune system and are associated with inflammatory symptoms. Both paediatric and adult patients face daily challenges related to their disease, diagnosis and subsequent treatment. For this reason, a survey was developed in collaboration between the FMF & AID Global Association and the Erlangen Center for Periodic Systemic Autoinflammatory Diseases. Methods: The aim of the survey was to collect the personal assessment of affected patients with regard to their current status in terms of diagnostic timeframes, the interpretation of genetic tests, the number of misdiagnoses, and pain and fatigue despite treatment. Results: In total, data from 1043 AID patients (829 adults and 214 children/adolescents) from 52 countries were collected and analyzed. Familial Mediterranean fever (FMF) (521/50%) and Behçet’s disease (311/30%) were the most frequently reported diseases. The average time to diagnosis was 3 years for children/adolescents and 14 years for adults. Prior to the diagnosis of autoinflammatory disease, patients received several misdiagnoses, including psychosomatic disorders. The vast majority of patients reported that genetic testing was available (92%), but only 69% were tested. A total of 217 patients reported that no increase in acute-phase reactants was detected during their disease episodes. The intensity of pain and fatigue was measured in AID patients and found to be high. A total of 88% of respondents received treatment again, while 8% reported no treatment. Conclusions: AID patients, particularly adults, suffer from significant delays in diagnosis, misdiagnosis, and a variety of symptoms, including pain and fatigue. Based on the results presented, raising awareness of these diseases in the wider medical community is crucial to improving patient care and quality of life.

## 1. Introduction

The term “autoinflammatory disease” was coined by McDermott et al. in 1999 and refers primarily to diseases of the innate immune system [1]. Most cases of autoinflammatory diseases are genetically classified as monogenic, while others are polygenic and multifactorial, caused by a variety of mutations. However, in more than half of individuals suspected of having an autoinflammatory disease, no causative gene was found [2]. Prior to genetic testing becoming widely available, patients were diagnosed solely on the basis of their clinical presentation. Many of these autoinflammatory diseases can mimic infectious, malignant, and rheumatic issues, resulting in a delayed diagnosis. Typically, medical survey data are collected by researchers for doctors. However, in this unique approach, patient experts (certified EUPATI—European Patients Academy on Therapeutic Innovation) and specialized physicians collaborated to develop this survey, aggregate the data, and collect the results. While participation was voluntary, respondents explicitly agreed to anonymized data analysis and the publication of results. The aim of this effort is to represent the value of the patients’ voices by capturing their responses and experiences in managing their AIDs and related symptoms.

Autoinflammatory diseases often begin in childhood or early adulthood and are frequently diagnosed many years after their onset [3]. These diseases may also appear later in life and are often not recognized in adult patients. Diagnostic delays can be attributed to a variety of factors. For example, a common misconception is that patients with familial Mediterranean fever (FMF) must be of Mediterranean descent or that autoinflammatory patients must have homozygous mutations to develop symptoms [4,5]. In addition, although autoinflammatory diseases often present as periodic fever syndromes, patients do not always have a fever or elevated acute-phase reactants. Due to the rarity of these diseases, it is understandable that medical knowledge is limited, and therefore, effective treatments are often initiated late or are not available. Colchicine is widely used in AIDs, including Behçet’s disease, as it reduces aphthous ulcers, controls inflammation, and reduces the intensity and frequency of relapses. Interleukin (IL)-1 inhibitors such as anakinra, canakinumab, and rilonacept are effective in the treatment of many different AIDs [6,7,8,9,10]. However, these biological drugs are not available in all countries, making effective disease control difficult [7,8,9,10,11,12,13,14,15,16,17].

Based upon these shortcomings and challenges, a survey was developed to investigate how these and other factors affect the diagnosis, treatment, and care of autoinflammatory disease patients.

## 2. Materials and Methods

### 2.1. Description of the Survey

A cross-sectional survey with 30 questions was developed by members of the patient organization FMF & AID Global Association (Executive Director, Malena Vetterli, and Research Officer, H. Ellen Cohen) under the guidance of Juergen Rech (MD, Head of the Centre for Periodic Systemic Autoinflammatory Diseases at the University of Erlangen-Nuremberg, Germany). For the purpose of the survey, the term uSAID (undifferentiated/undiagnosed systemic autoinflammatory diseases) is used when a patient has an unidentified autoinflammatory disease. The survey was first published at the beginning of 2021, was available for 6 weeks on the EU-Survey platform from the European Commission, and was shared on social media in Facebook closed groups. The survey was translated from English into German, French, Italian, Spanish, Portuguese, Turkish, Arabic, Greek, Hebrew, Russian, and Georgian. The objective of the survey was to collect data on the given diagnoses (FMF, periodic fever, aphthous stomatitis, pharyngitis, and adenitis (PFAPA) syndrome, familial cold autoinflammatory syndrome (FCAS), Muckle–Wells syndrome (MWS), neonatal-onset multisystem inflammatory disease and others). Chronic infantile neurological cutaneous and articular syndrome (NOMID/CINCA), TNF receptor-associated periodic syndrome (TRAPS), Mevalonate kinase deficiency (MKD), familial cold autoinflammatory syndrome-2 (FCAS2), NOD2-related systemic autoinflammatory granulomatosis (Blau Syndrome), Yao syndrome (NOD2-associated autoinflammatory disease, presents with erythematous plaques and patches, periodic fevers, myalgia, gastrointestinal (GI), and sicca-like symptoms), deficiency of adenosine deaminase 2 (DADA2), Behçet’s disease, unspecific systemic autoinflammatory disease (uSAID), patient access to specialists, genetic testing, diagnostic time frames, misdiagnoses, access to treatment, patient satisfaction, pain management, level of fatigue and patient wellbeing. In addition, it was shared with organizations affiliated with FMF & AID (see Acknowledgments section) and collaborating groups, with a total of approximately 100,000 patients worldwide. Participation in this survey was voluntary, and all those who took part agreed that the anonymized information from the survey could be analyzed and published. This study was approved by the International Review Board of the University Clinic Erlangen (#362_20 Bc).

### 2.2. Statistical Analysis

Statistical analyses were essentially descriptive and exploratory. Summary statistics were calculated based on the scales of the characteristics, i.e., categorical variables were summarized using counts and percentages, and scale variables were summarized using means and standard deviations. Data manipulation and visualization were undertaken using Excel.

## 3. Results

The survey dataset results indicated the average time to diagnosis in children/adolescents was 3 years and 14 years for adults. Patients received multiple misdiagnoses prior to a correct AID diagnosis, including psychosomatic disorders (210 patients), fibromyalgia (140 patients), osteoarthritis (69 patients), irritable bowel syndrome (200 patients), and asthma (78 patients). The vast majority of patients reported that genetic testing was available (92%), while only 69% were tested. A total of 217 patients reported that no elevation of acute-phase reactants was found during their flares. The intensity of pain and fatigue were measured among AID patients and found to be high. A total of 88% of respondents received treatment, while 8% reported no treatment.

### 3.1. Demographic Characteristics

**Figure 1:**
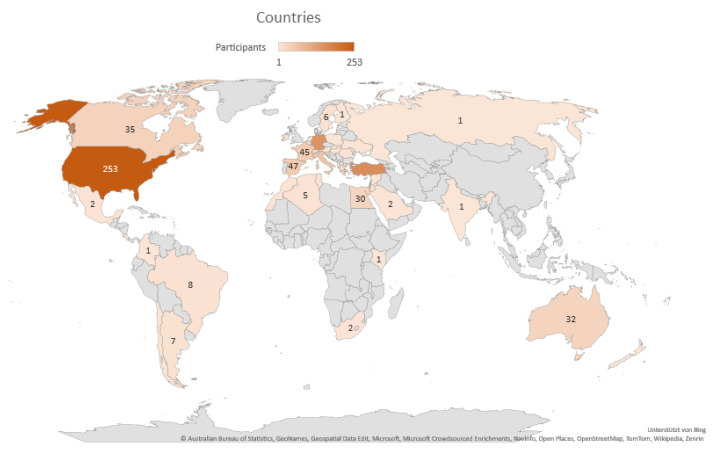
Indication of countries and the number of participants per country who completed the questionnaire.

FMF & AID received 1043 responses from patients who completed the survey (Figure 1). A total of 829 patients (79.48%) were adults (>18 years) and 214 children/adolescents (20.51%). AID patients from many different countries participated in this survey, with the majority of responses coming from patients in the USA, Turkey, and Germany. A wide spectrum of AID patients contributed to this survey, with FMF and Behcet’s disease being the most frequent diagnoses. The average timeframe for a child/adolescent to be diagnosed with AID was 3 years, while the average timeframe for an adult patient was 14 years. While pediatric patients are diagnosed in a shorter timeframe than adults, parent and caregiver satisfaction was extremely low.

### 3.2. Genetic Testing

**Figure 2:**
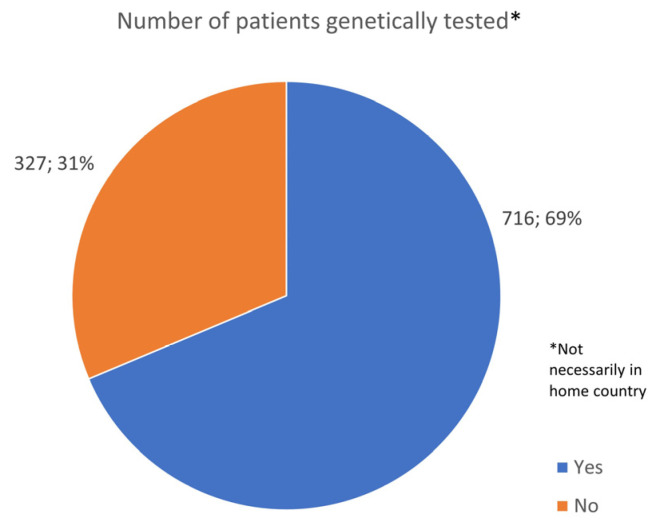
Percentage of participants who had or did not undergo genetic testing.

**Figure 3:**
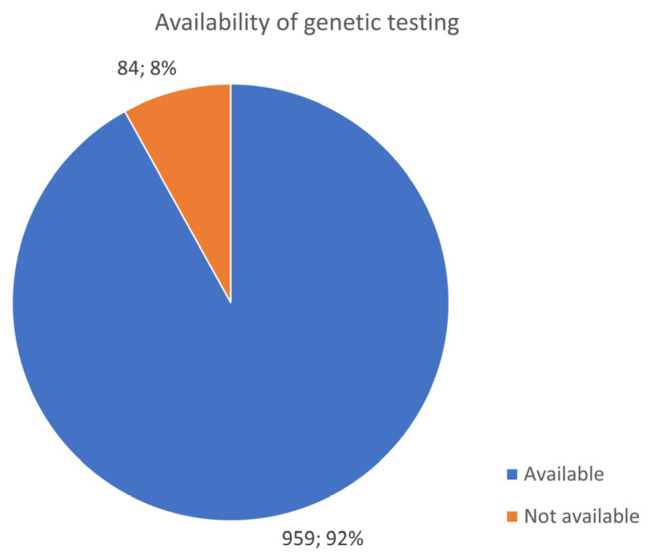
Basic availability of genetic testing overall across all countries of the participants in the survey.

The vast majority of patients reported that genetic testing was available (92%) and was carried out (69%) (Figure 2 and Figure 3). Insurance paid for genetic testing in 56% of cases, while 19% required self-payment, and 6% reported a mixed payment model. Of note, not all tested patients had access to their genetic results, and other patients were denied genetic testing.

### 3.3. Laboratory Changes

A total of 217 (20.81%) patients in this survey reported that inflammatory markers in blood tests, such as C-reactive protein (CRP), erythrocyte sedimentation rate (ESR), or serum amyloid A (SAA), were not elevated during a disease flare-up. Of note, SAA testing is not available in the United States.

### 3.4. Disease Impact on Pain and Fatigue

**Figure 4:**
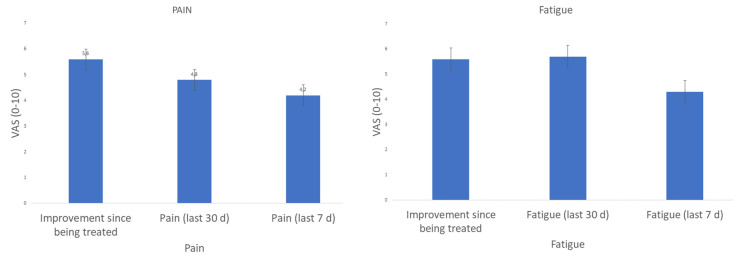
Display of pain and fatigue intensity 7 days (7 d) and 30 days (30 d) retrospectively, as well as the improvement in the entire period from the beginning of treatment to the present.

The intensity of pain associated with AID was measured using the standard visual analog scale (VAS 0–10). Patients were asked to rate their pain over the last 7 and 30 days. The patient feedback indicated substantial pain levels with a mean VAS of 4.2 (SD +/−3.0) during the last 7 days and 4.8 (SD +/−2.7) during the last 30 days (Figure 4). 

The intensity of fatigue was measured by VAS (VAS 0–10). Also, the fatigue burden was high, with a mean VAS of 5.3 (SD +/−3.0) during the last 7 days and 5.7 (SD +/−2.8) during the last 30 days (Figure 4). 

Over-the-counter medications, such as acetaminophen, metamizole, and aspirin, as well as prescribed NSAIDs, such as ibuprofen, diclofenac, and naproxen, were used for pain control. Some patients also required opioids and neuropathic pain medications, such as pregabalin and gabapentin. A total of 88% of respondents received treatment, 8% did not receive treatment, and data were not available for 4% of participants.

### 3.5. Monogenic Patients Presenting with a Fever

A total of 690 patients with monogenic disease responded to the survey, and surprisingly, a marked difference should be noted between children and adults expressing fevers as a clinical symptom in their individual disease presentation.

**Figure 5:**
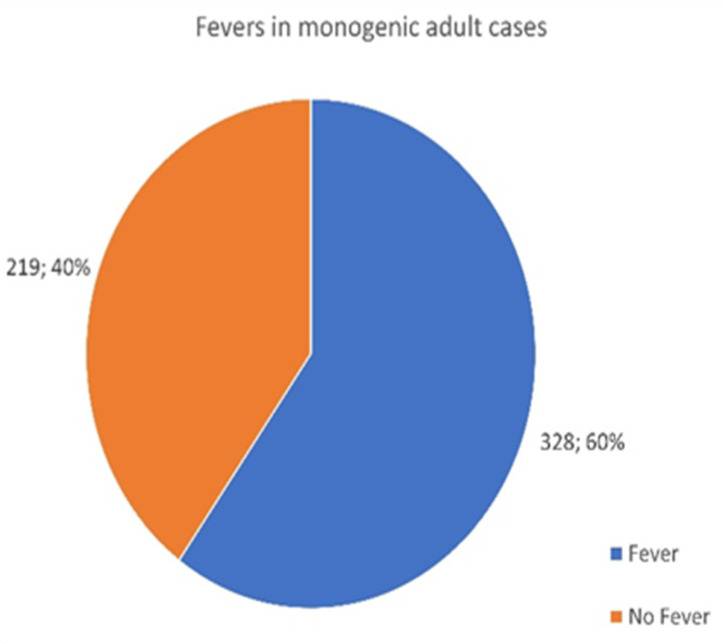
Number of adult patients who reported episodes of fever associated with their monogenic disease.

**Figure 6:**
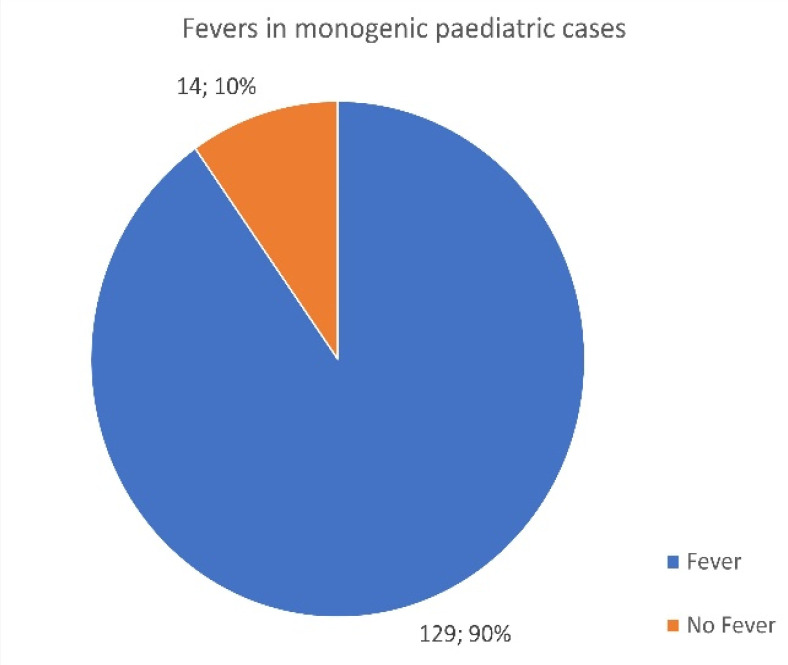
Number of pediatric patients who reported episodes of fever associated with their monogenic disease.

Of the 547 adults identified, only 328 reported fevers as an initial disease presentation (Figure 5), whereas out of the 143 pediatric cases reported, 129 presented with a fever (Figure 6). While fevers are often an assumed factor in AID, they are not always present in all-age patients. Adults appear to present with fewer fevers than children. A total of 40% of adult respondents were absent from fevers, compared to only 9.8% of children. 

This significant difference raises issues as to how the aging process impacts body temperature in these innate immune responses over time and how unknown metabolic pathways or genetic variants alter the presentation of fevers from disease inception into adulthood.

It is important to note that 152 all-age patients indicated low or hypothermic temperatures (between 34 °C/93.2 °F and 36 °C/96.8 °F) present during flare times, and further medical investigation should be undertaken to not only better understand the mechanics of body temperature in these disorders but to also ensure that these patients are not incorrectly dismissed or ruled out of an AID diagnosis. Additionally, it is critical for adult AID-treating physicians to carefully review and note if childhood fevers in these older populations were a prior factor in their case work-up.

### 3.6. Monogenic Patients Presenting with Neurological Symptoms

**Figure 7:**
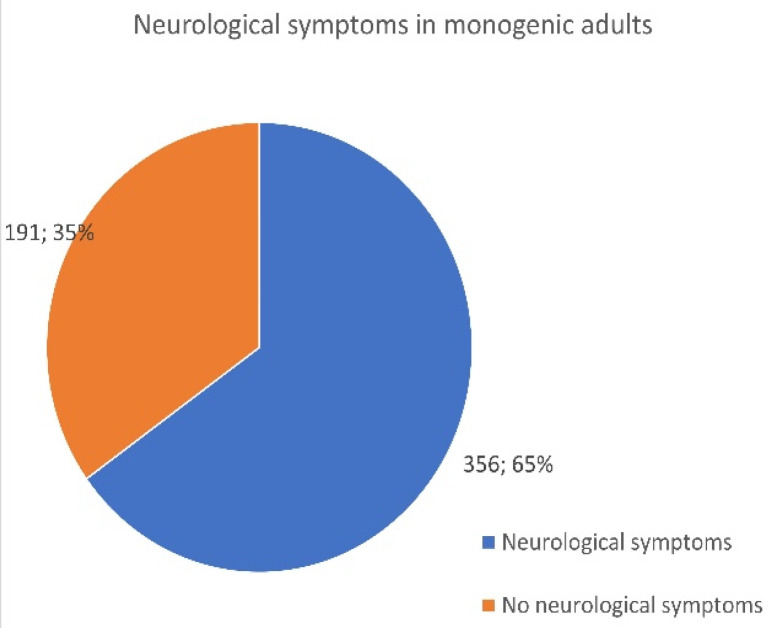
Number of adult patients who reported neurological problems related to their monogenic disease.

**Figure 8:**
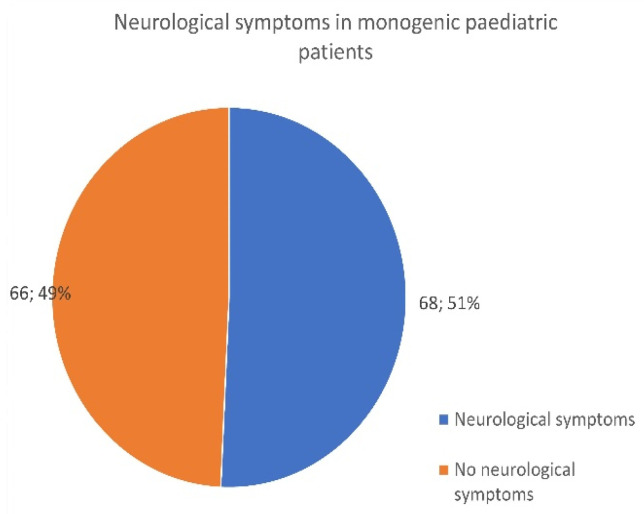
Number of child patients who reported neurological problems related to their monogenic disease.

Neurological impacts were also noted in 424 AID monogenic children and adults. Symptoms, including headaches, seizures, bad memory, brain fog, and lack of concentration, were reported by 356 adults (Figure 7) and 68 patients under the age of 18 (Figure 8).

While there is newfound awareness as to the importance of AID inflammation impacting the brain and central nervous system (CNS), it will be important for all age patients to be assessed and managed, as these neurological issues may change or emerge over time. A higher percentage of adults (65%) reported having problems per the survey as compared to 51% of children.

### 3.7. Issues in Diagnosis and Disease Management

**Figure 9:**
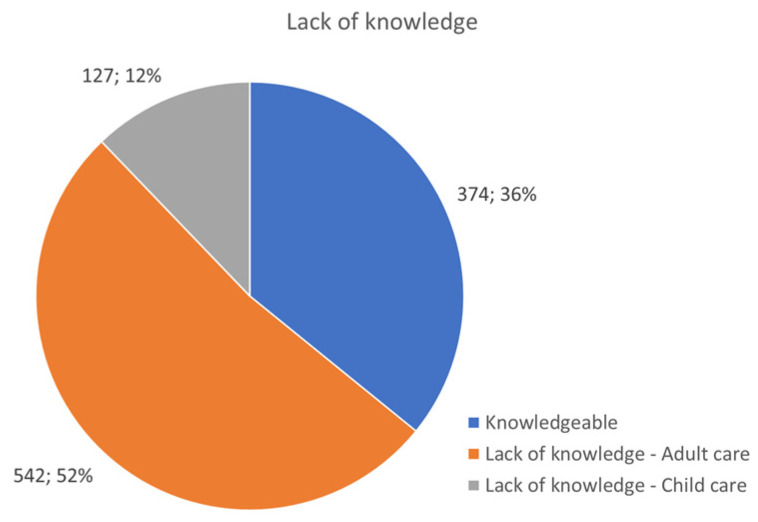
Lack of medical knowledge and experience was reported by patients.

**Figure 10:**
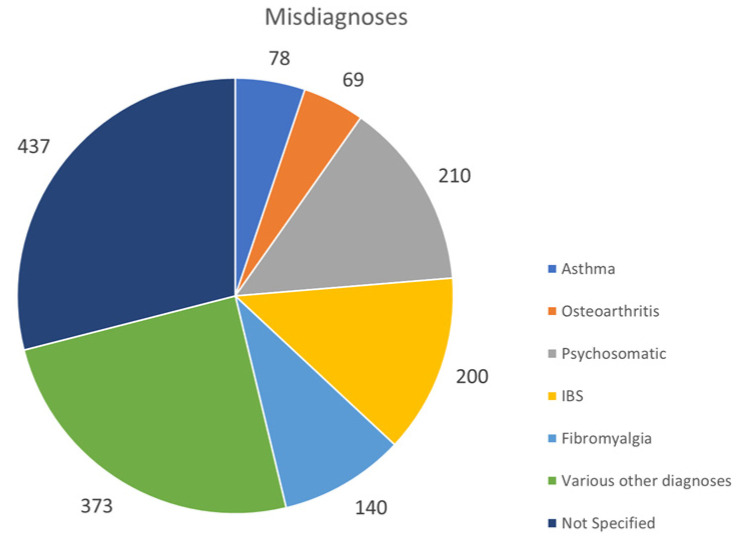
Frequency and designation of incorrect diagnoses.

A total of 669 AID patients (64.14%), 542 of whom were adults, and the remaining 127 children/adolescents considered that their medical team lacked specific knowledge and experience to appropriately diagnose, treat, and monitor AID (Figure 9). A total of 271 AID patients, among them 243 adults and 28 children/adolescents, reported being dismissed, contributing to a delay in care. Misdiagnosed conditions included psychosomatic disorder (210 patients), fibromyalgia (140 patients), osteoarthritis (69 patients), irritable bowel syndrome (200 patients), and asthma (78 patients) (Figure 10). The most important barriers patients experienced were not being taken seriously with regard to their symptoms, refusing appropriate and timely blood testing during flares, encountering physicians unwilling to consult with experts in the field, or rejecting the case outright.

### 3.8. Medical Treatment in AID

**Figure 11:**
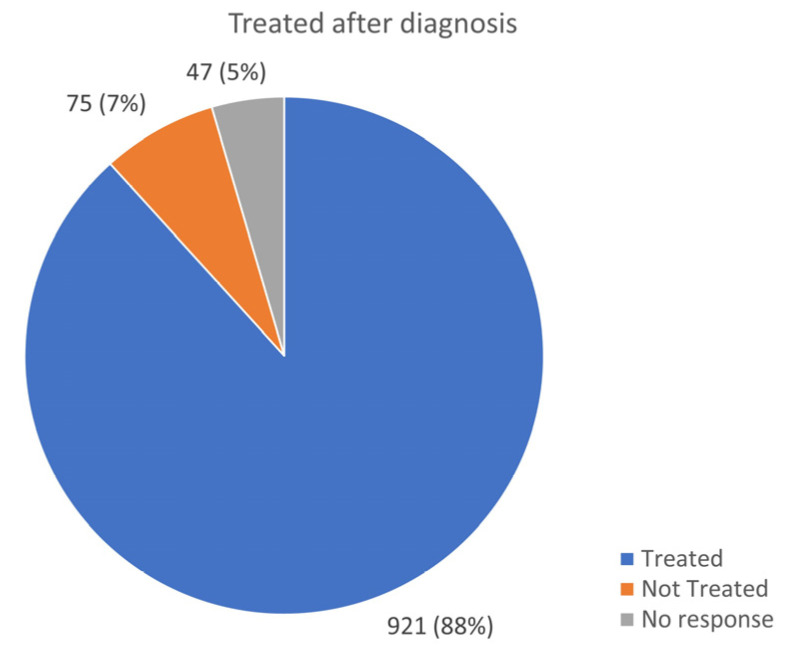
Number of patients treated after diagnosis.

**Figure 12:**
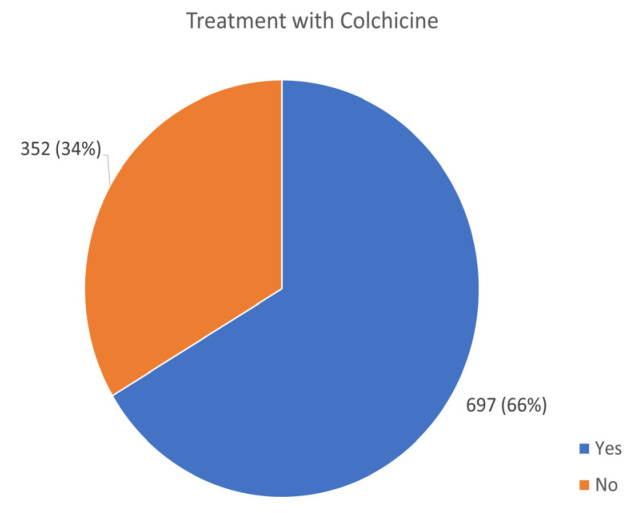
Number of patients treated with colchicine.

The majority of patients (921 patients; 88%) responded that their AID was treated after being diagnosed (Figure 11). A total of 697 (66%) patients were put on drug treatment. Colchicine, which is the first line of treatment for FMF, is also widely used for PFAPA, Behcet’s, uSAID, CAPS, TRAPS, and HIDS. It was the most frequently used drug cited in this survey (Figure 12). 

Biological drugs such as IL-1 inhibitors (anakinra, canakinumab, and rilonacept), IL-6 inhibitors (tocilizumab), and TNF-alpha blockers (i.e., infliximab, adalimumab, and etanercept) are also used for AID treatment. Conventional immune-modulating medications such as prednisolone, azathioprine, methotrexate, and hydroxychloroquine were also reportedly used.

### 3.9. IL-1 Biologic Use in Pediatrics and Adults—Quality of Life Improvement

**Figure 13:**
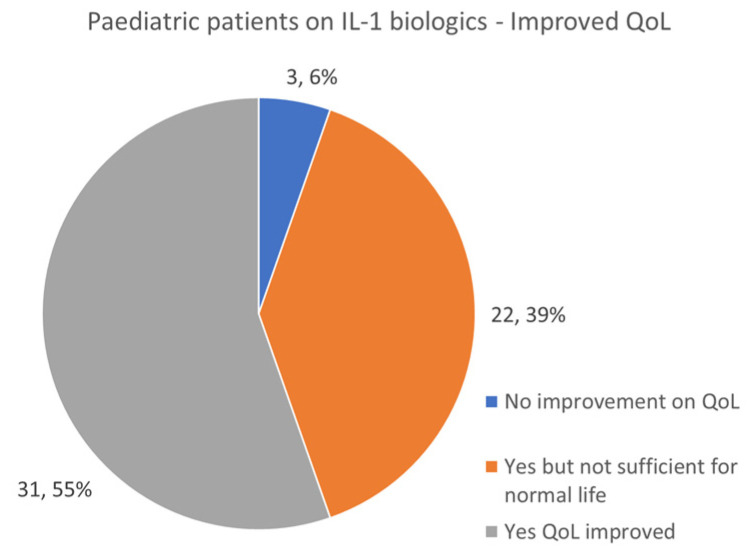
Number of pediatric patients receiving IL-1 biological therapy and Quality of Life (QoL) response.

**Figure 14:**
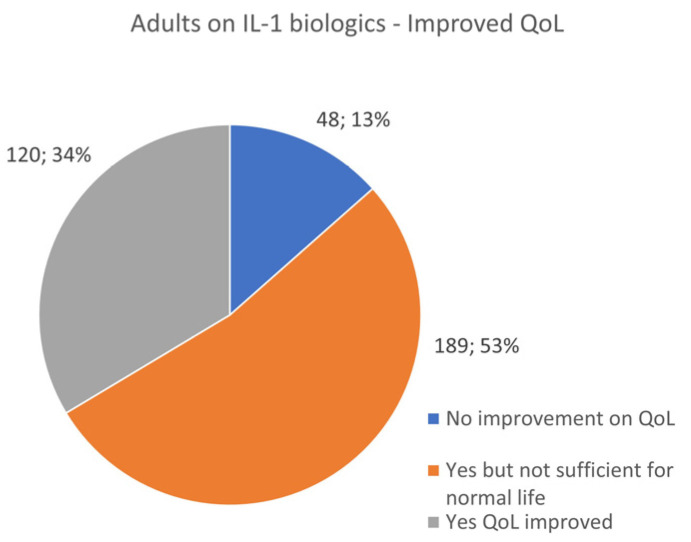
Number of adult patients receiving IL-1 biological therapy and Quality of Life (QoL) response.

According to survey responses, IL-1 inhibitors had different quality of life (QoL) success rates between children and adults. While 55% of pediatric patients reported QoL improvement on IL-1 biologic treatment, a surprising 39% only had a partial resolution, which did not translate into a robust and symptom-free QoL. A total of 6% reported no QoL improvement on IL-1 medications (Figure 13). Whereas 53 percent of adult patients reported that using IL-1 biologics did not provide enough efficacy for full QoL, only 34% regained full functionality. A total of 13% reported no improvement with medication use (Figure 14).

These data raise a variety of concerns related to the overall efficacy of IL-1 inhibitors, implications from the medical literature reporting full symptom resolution based on small samples of rare patients, and equal tolerability of these medications in all age populations.

Patients who do not make a full resolution on these IL-1 inhibitors should be considered for an increased dose of medication or for prescribing a secondary biologic for better symptom control. Despite the use of biologics, even at higher doses, patients may present with infrequent breakthrough flares and require additional medications, including steroids and pain control.

### 3.10. Treated Patients Recording a 5+ Pain Score

**Figure 15:**
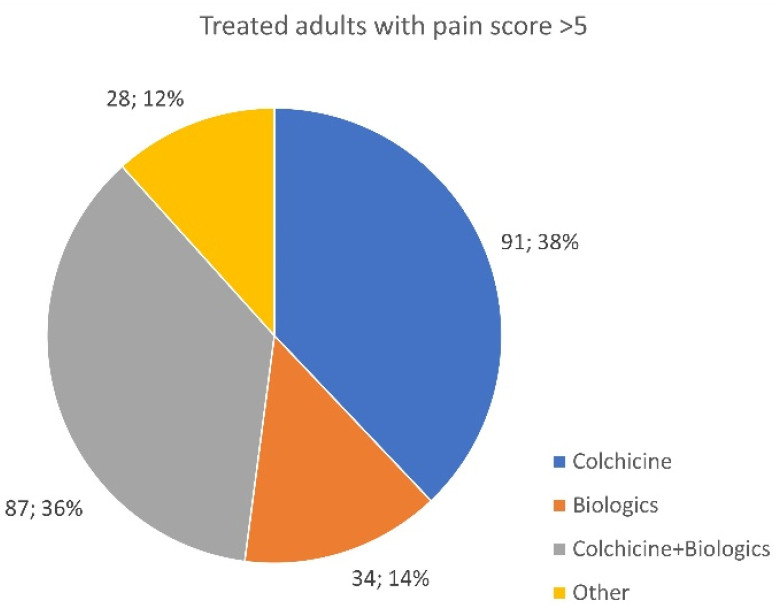
Distribution of applied therapies in adult patients with a monogenic disease and a pain score assessment according to the visual analogue scale > 5 (norm 0–10).

**Figure 16:**
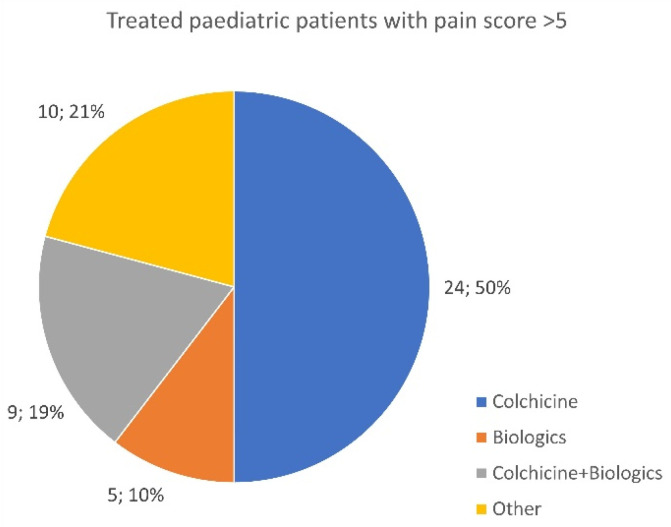
Distribution of applied therapies in pediatric patients with a monogenic disease and a pain score assessment according to the visual analogue scale > 5 (norm 0–10).

Despite treatment, 468 out of 1043 respondents reported having a mean pain score of 5+ or above in the last 30 days (pain score: 0 “no pain”—10 “as bad as it could possibly be”). Out of these 468 patients, 288 diagnosed with a monogenic disease (240 adults (Figure 15) and 48 children (Figure 16)) experienced a pain score of 6 or above despite being treated with colchicine, biologics, or a combination of both. 

The survey data indicate that 44.9% of treated patients have high levels of pain, which must be taken seriously by treating physicians. With almost half of patients reporting elevated pain scores, treatment protocols should be revised and further researched. Dosing adjustments of both colchicine and biologic medications should be reconsidered based on patient symptoms and pain response, despite negative APR results. Patients on colchicine, experiencing pain, or those on the highest safe doses should be considered for a biological drug addition if medication is available, regardless of cost. 

Finally, it is important to change the perception that colchicine and/or biologics provide complete resolution of all disease symptoms, as there is no cure for autoinflammatory diseases. Additional medication options must be incorporated based on the treated patient’s lived experience. It should be recognized that pain management in autoinflammatory diseases may not always be effective, as some patients do not respond to or tolerate NSAIDs and may require opioids or corticosteroids to control breakthrough symptoms and pain. These add-on treatments should be considered the standard of care for all those managing uncontrolled symptoms.

### 3.11. Global Distribution of Familial Mediterranean Fever Cases

Familial Mediterranean fever (FMF) is a monogenic disorder and the most common inherited autoinflammatory disease. The disease presentation is more prevalent in people of Mediterranean descent. However, patients with FMF have been identified globally. It equally affects males and females, usually presenting before 20 years of age. Symptoms include abdominal pain, recurring fevers, joint pain and swelling, headaches, pharyngitis, ankle swelling, monoarthritis, chest pain, pericarditis, leg pain, myalgia, cutaneous manifestations (erysipelas-like erythema, non-specific purpuric rash, Henoch–Schönlein purpura), orchitis, fatigue and others.

**Figure 17:**
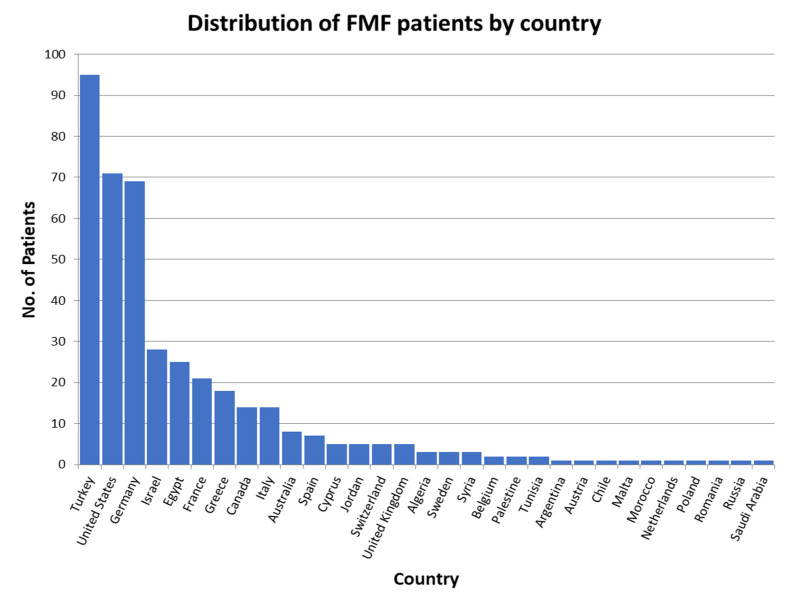
Worldwide distribution of Familial Mediterranean fever (FMF) patients per country who participated in the survey.

The survey was completed by 415 patients who were diagnosed with FMF and without other co-existing autoinflammatory diseases. Patients with FMF included 70 children/adolescents and 345 adult cases. Respondents from 31 countries diagnosed with FMF were noted in the survey, with Turkey having the highest number with 95 cases, followed by the United States with 71 cases, and Germany with 69 cases (Figure 17). FMF is diagnosed worldwide.

### 3.12. FMF Patients QoL Improvement after Treatment

A total of 415 all-age FMF patients reported on QoL after treatments were used, including colchicine, anakinra, canakinumab, Humira, Enbrel, and tocilizumab.

**Figure 18:**
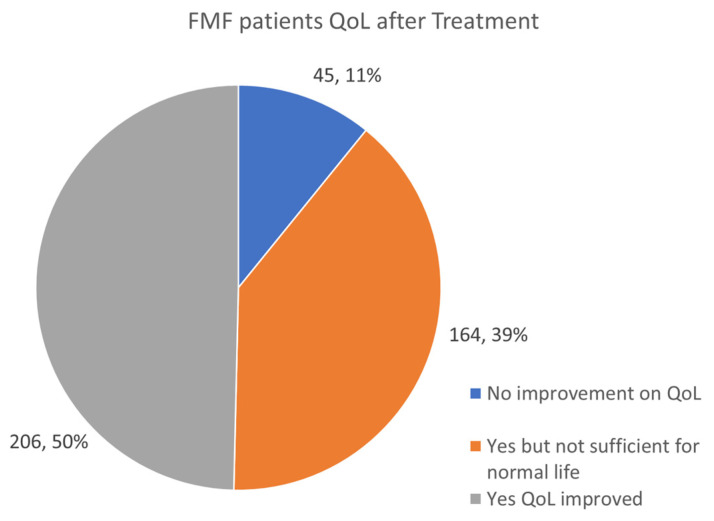
Assessment of the response to therapy and improvement of QoL in FMF patients.

Despite treatment, only 206 patients reported an improved QoL, while 164 reported that, although medicated, their QoL was still compromised. A total of 45 patients reported that, with treatment, QoL had not improved (Figure 18). FMF is a complex autoinflammatory disease, and despite the global use of colchicine, it does not provide 100% symptom relief for all patients. Additionally, IL-1 inhibitors, used with or without colchicine, do not always provide symptom resolution. 

QoL for FMF patients remains challenging, and survey responses raise important questions with regard to the dosing of medications and the overall efficacy of limited treatments. It is critical in the future for medications to be developed targeting the inflammatory pathways unique to FMF patients.

### 3.13. Global Distribution of Behçet’s Cases

Behçet’s disease is a polygenic disorder that likely developed along the ancient “silk road” and impacted people of Mediterranean and Asian descent. This vascular-driven autoinflammatory disease typically presents more severely in males and involves a constellation of symptoms, including mouth/genital ulcers, uveitis/inflammatory eye issues, skin pustules, GI ulcerations, neurological problems, joint pain, and various-size vessel vasculitis.

**Figure 19:**
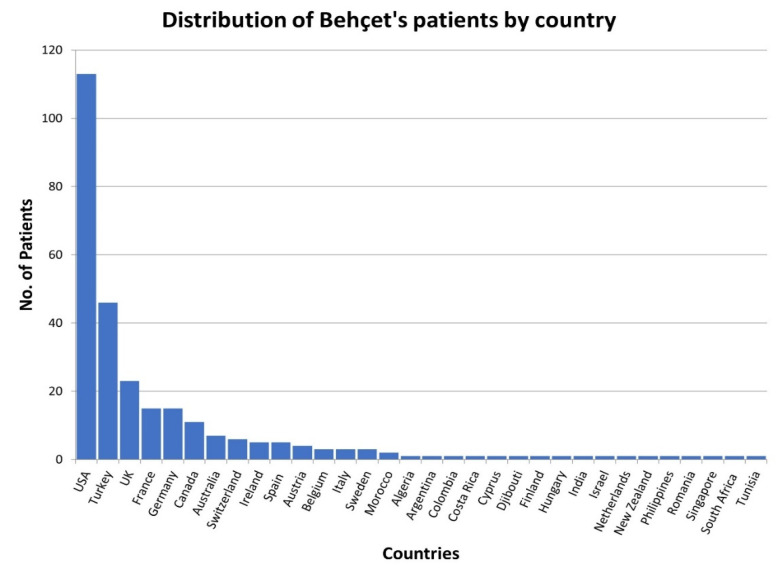
Worldwide distribution of the number of Behçet patients per country.

The survey was completed by 278 Behçet patients (8 children/adolescents and 270 adults). The location of patients responding suggests that the disease manifests perhaps more globally than current medical literature suggests (Figure 19). Respondents from 32 countries diagnosed with Behçet’s were noted in the survey, with the USA having the highest number with 113 cases, followed by Turkey with 46 patients. Further research is needed to determine the actual prevalence of Behçet in all countries and all sections of the population. 

Despite the country of origin where the symptomatic patient resides, it is important that treating physicians do not dismiss these cases with the incorrect assumption that the location of the patient is the overarching criteria for a Behçet’s diagnosis. Cases continue to be identified in Europe, Australia, the United States, South America, and others beyond the Middle East and Asian regions.

### 3.14. Improving the Quality of Life of Behcet Patients after Treatment

A total of 278 all-age Behçet (polygenic) patients reported on QoL after treatments including colchicine, apremilast, baricitinib, tofacitinib, cyclosporine, azathioprine/Imuran, methotrexate, infliximab, etanercept, adalimumab, secukinumab, tocilizumab, ustekinumab, anakinra, rituxan, interferon, golimumab, certolizumab pegol, and IVIG replacement. 

**Figure 20:**
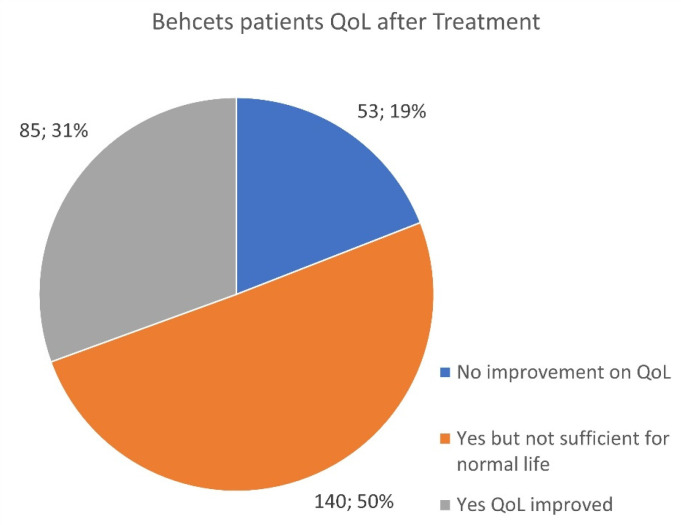
Assessment of the response to therapy and improvement of QoL in Behçet patients.

Despite a wide variety of treatments utilized, QoL was reported to be compromised in 69% of these patients (Figure 20). Behçet’s is a complex multi-organ disease that impacts a wide variety of body systems and presents differently in each case. Treatment decisions are often made based on affected organs and tissues. For this reason, the list of possible drugs is larger. 

Regardless of the various treatments used, concerns about QoL remain challenging and raise issues as to the dosing efficacy of listed medications, unknown genetic factors impacting symptom control, and other physiologically affected pathways that have yet to be acknowledged or discovered. 

## 4. Discussion

Autoinflammatory diseases are genetically based disorders associated with a dysregulation of the innate immune system, resulting in uncontrolled inflammation [17]. Variants associated with AIDs are included in the 2022 classification of human inborn errors of immunity [18], many of them caused by monogenic germline or somatic mutations, and include 485 distinct disorders categorized into autoimmune, autoinflammatory, allergic, and malignant phenotypes [19]. The most well-known AID is familial Mediterranean fever (FMF), which is caused by variants in the MEFV gene [20]. It is known to be highly prevalent in the eastern Mediterranean region. However, with advances in genetic screening and migration, the diagnosis of FMF patients has markedly increased across all countries [21,22,23,24,25,26,27,28,29,30,31,32,33,34,35,36,37]. AIDs are often severe conditions that require specialized care but are not readily recognized. They usually affect patients early in their lives and will accompany them for a lifetime. Proper management is of paramount importance. This survey, with over a thousand responses, provides an overview of patients’ experiences related to the diagnosis and management of AID. 

Familial Mediterranean fever and Behcet’s disease were the most common diseases reported in the survey. While both may present in higher population numbers within the Mediterranean and Asian regions, it is critical that medical professionals globally recognize that each disease is prevalent across numerous countries and affects all age patients [20,38,39]. The survey responses also highlight the poor QoL in FMF and Behcet’s patients despite treatment, which can be attributed to the complexity of these rare diseases and the lack of specialized medical knowledge.

While diagnosis in pediatric patients is relatively timely, adults with AID are often misdiagnosed and not treated. This is due to overlapping symptoms mimicking infections, skin diseases, malignancies, gastrointestinal diseases, allergic conditions, and immune deficiencies. It is also common for AID patients to be diagnosed with autoimmune diseases such as Sjögren’s syndrome, rheumatoid arthritis, and systemic lupus erythematosus, with detrimental impacts on the patient’s health due to a delayed diagnosis and incorrect treatment. Additionally, since AIDs are rare diseases that are not routinely included in medical curricula, physicians may not be aware of them. As a consequence, patients are often not listened to, not taken seriously, and even ignored, which requires them to seek several consultations until they can locate a provider who has the necessary experience, knowledge, and expertise to diagnose and manage their AID [31,32]. 

Recent advancements in molecular research on these diseases have resulted in a more accurate classification that will continue to grow as new genetic mutations are identified. In 2002, Infevers [40] was developed as a database tool for doctors and clinicians to verify genetic data for AIDs. The aim of this international registry is to collect and report demographic, genetic, and clinical data for all currently known monogenic autoinflammatory disorders. Nevertheless, there is a high percentage of patients who have symptoms but have not yet received a proper diagnosis. These patients may be overlooked due to being uSAID, lacking specific inflammatory markers, or having unclassified genetic variants. The survey revealed that although genetic testing is performed frequently, patient access to full results (all mutations found, including benign) is still suboptimal, and inadequate interpretation may be an obstacle. Additionally, reimbursement or insurance coverage for genetic testing remains challenging. 

The survey also reveals that pain is one of the predominant causes of disability in AID [41,42,43,44,45]. Only 10% of patients reported they were pain-free. Half of the survey respondents indicated a pain score of more than 4 on a 0–10 scale, reflecting inadequate pain control despite the use of medication. Pain is often poorly understood in AID, although there is increasing evidence that the immune system may play a role in the development of pain. 

Additionally, fatigue is a major component of AID and is described by patients as profoundly debilitating, impacting every aspect of their lives [38,39,45,46,47,48,49]. Consequently, fatigue may severely affect wellbeing, causing a financial burden for the individual, family, and society. In childhood/adolescence, it affects school attendance and performance, socialization, and participation in sports or physical activities. In adults, the fatigue may be disabling and impacting the overall quality of life, including career/work, relationships, family/parenting, and self-esteem.

Moreover, it is vital for each age group to be appropriately assessed and treated by neurologists for the medical impacts of headaches and seizures. However, it is also important to consider that all-age patients expressing symptoms be cognitively evaluated by a neuropsychology specialist for executive function deficiencies, short/long-term memory retrieval, educational disabilities, ADHD, etc. These types of evaluations and findings will ensure that learning and workplace productivity can be optimized by incorporating accommodations and treatments as needed, thus allowing for robust patient functionality [45,47,48,49].

It is also relevant to ensure that cognitive issues in children are addressed by caregivers, as pediatric patients often do not have the language abilities to express struggles with complex processing issues. Assessment and capture of each individual’s problems will be important to allow for necessary support in schools and other educational environments. 

In rheumatic diseases, the association between fatigue and pain has been well documented. Fatigue is often associated with increased pain, and both can be synchronous [38,39,46,47,48,49,50,51]. Inflammatory mechanisms related to the etiology of fatigue implicate a significant involvement of cytokines. Interleukin (IL)-1 beta (IL-1β), tumor necrosis factor alpha (TNF-α), IL-6, and interferon gamma (IFN-γ) are mediators that can induce fatigue. Cytokines regulate normal physiological functions, including mood, cognition, and sleep, and their expression varies over the course of the day and in response to local activity. Consequently, it is likely that dysregulation of inflammatory cytokines in AIDs can contribute to fatigue [46,52,53,54,55,56,57,58,59,60,61,62]. 

Autoinflammatory diseases often present with recurring attacks of fever, abdominal pain, arthritis, and skin rashes. These symptoms are often tied to elevated acute-phase reactants [50]. This survey also revealed that testing for the elevation of inflammatory markers is not often attainable for patients during flare periods. Testing for serum markers of inflammation is not always available or ordered by physicians. Isolated CRP testing may not reveal the entire molecular spectrum of inflammation in AID patients. Additionally, expectations of having extremely elevated inflammatory markers while ignoring mild elevations or subclinical changes, including symptoms, may lead to misconceptions about the presence of an AID [35]. Also, acute-phase reactants may not be present in each individual AID entity [35]. 

Colchicine is the first-line treatment for FMF [3,62]. It is often used in other AIDs such as PFAPA, Behcet’s disease, and uSAID. This medication is particularly useful for treating oral and/or genital lesions and significantly reduces inflammation while increasing the symptom-free interval between flares [20]. Colchicine is an inexpensive and safe drug and the most frequently used medication cited in this survey. There are differences among the colchicine brands. Individual patients may tolerate or respond differently to the various preparations [7,8,9,10,11]. However, certain patients may not tolerate or respond effectively to colchicine and, therefore, require alternative treatments such as IL-1 targeting agents [13,14,15,16]. Patients requiring biological drugs often encounter accessibility challenges, such as physicians refusing to prescribe medications due to high costs, insurance companies not approving biological medications, limited approval (only a small number of patients are allocated to receive medication), or the drugs not being available in the patient’s country of residence. 

Beyond prescribing issues, in some cases, patients may refuse biological treatment due to a fear of not understanding how the medication works, concern about lifelong medication use, and possible adverse reactions. Thus, doctors may require extended time with patients to explain why the medication is being prescribed, common side effects, and how to manage potential reactions. These issues may change in the future due to new and promising therapeutic approaches, which are currently in either study phases or in development. These include inhibiting the NLRP3 inflammasome and small-molecule inhibitors that impact signaling pathways either upstream or downstream of the inflammasome [57]. 

Doses of biologics for controlling AID may vary. For example, CAPS (Cryopyrin-associated periodic syndrome) patients may require higher or more frequent doses of IL-1 biologic medications [13]. Inadequate or suboptimal dosing of IL-1 drugs for autoinflammation may lead to underreporting of medication efficacy. 

There were limitations in this study. Respondents were not required to submit laboratory reports or medical records. Additionally, both monogenic and polygenic autoinflammatory diseases were included. Although the diagnosis of these participants could not be confirmed via medical records, there would be little purpose for patients or their parents to allot time to respond to the survey questions, as the link was only shared with those in autoinflammatory-specific and private social media groups. 

This study had the largest global response of pediatric and adult patients providing feedback regarding their disease status. Over a thousand responses support the data presented in this paper.

## 5. Conclusions

The survey results ascertain that autoinflammatory patients of all ages are burdened in many ways by not receiving a timely or correct diagnosis. Patients, per their survey responses, often struggle through multiple misdiagnoses, incorrect treatments, and a lack of specialized medical care, as well as contend with a compromised quality of life. The most common misdiagnoses reported included asthma, osteoarthritis, psychosomatic, IBS, fibromyalgia, etc. The patient ordeal is reflected as adults are noted to have longer diagnostic delays of, on average, 14 years vs. 3 years for most pediatric cases.

These delays are attributed to a lack of both medical knowledge and specialized autoinflammatory care. Genetic testing is an important tool for AID diagnosis. Unfortunately, it is not available in every country. Additionally, genetic criteria may be absent despite symptoms, emphasizing the importance of patients receiving a clinical diagnosis of uSAID to access treatment. 

Biomarkers for these diseases require further discovery, as many patients in our survey reported having negative acute-phase reactants (APR) during disease flare-ups, which are considered a hallmark of AID. Lack of elevations in APR delays diagnosis and treatment, prolonging the patient’s pain and suffering. Worldwide availability and low cost make colchicine a commonly prescribed drug for the initial treatment of AID, while biological agents were also used, according to the survey results. However, treatment with either colchicine or IL-1 biologics does not guarantee that patients will be asymptomatic and have full disease resolution. As our survey indicates, patients’ pain is a common finding, and medications used include NSAIDs, opioids, and others. 

This unique collaboration between FMF & AID Global Association and their medical partners in Erlangen enabled the development and execution of this study. This model of relationship-building between patient organizations and dedicated autoinflammatory centers is unique and allows for the identification of critical patient diagnostic shortfalls, gaps in treatment efficacy, and quality-of-life issues impacting all-age patients. These important factors are key to guiding therapy and developing new drugs. In the future, it will be critical to include the voice, experience, and expertise of these rare autoinflammatory patients prior to planning research initiatives and clinical trials. Patients and their families living with AID carry a significant social, financial, and medical burden that requires comprehensive support and care. Efforts must be made to strengthen physicians' and medical professionals’ knowledge by providing continuing medical education opportunities, specialized training courses from AID expert centers, and an early interventional curriculum on AID for medical students. Collectively, patient organizations, treating physicians, researchers, health authorities, and medical institutions need to work together in partnership to improve the lives of AID patients. 

## Data Availability

Raw data were generated at FMF & AID Global Association, www.fmfandaid.org, Zurich, Switzerland. Derived data supporting the findings of this study are available from the corresponding author (JR) upon request.
